# Diversity of Nicotinic Acetylcholine Receptor Positive Allosteric Modulators Revealed by Mutagenesis and a Revised Structural Model[Fn FN3]

**DOI:** 10.1124/mol.117.110551

**Published:** 2018-02

**Authors:** Joseph Newcombe, Anna Chatzidaki, Tom D. Sheppard, Maya Topf, Neil S. Millar

**Affiliations:** Departments of Chemistry (J.N., T.D.S.) and Neuroscience, Physiology and Pharmacology (A.C., N.S.M.), University College London, London, United Kingdom; and Institute of Structural and Molecular Biology, Birkbeck, University of London, London, United Kingdom (J.N., M.T.)

## Abstract

By combining electrophysiological and computational approaches we have examined a series of positive allosteric modulators (PAMs) acting on the human *α*7 nicotinic acetylcholine receptor (nAChR). Electrophysiological studies have focused on three *α*7-selective PAMs (A-867744, TBS-516, and TQS) that display similar effects on wild-type *α*7 nAChRs. In addition to potentiating agonist-evoked responses, all three compounds reduce receptor desensitization and, consequently, are classed as type II PAMs. Despite having similar effects on wild-type receptors, A-867744 was found to have profoundly differing effects on mutated receptors compared with TBS-516 and TQS, a finding that is consistent with previous studies indicating that A-867744 may have a different mechanism of action compare with other *α*7-selective type II PAMs. Due to evidence that these PAMs bind within the *α*7 nAChR transmembrane region, we generated and validated new structural models of *α*7. Importantly, we have corrected a previously identified error in the transmembrane region of the original cryo–electron microscopy *Torpedo* model; the only pentameric ligand-gated ion channel imaged in a native lipid membrane. Real-space refinement was used to generate closed and open conformations on which the *α*7 models were based. Consensus docking with an extended series of PAMs with chemical similarity to A-867744, TBS-516, and TQS suggests that all bind to a broadly similar intersubunit transmembrane site. However, differences in the predicted binding of A-867744, compared with TBS-516 and TQS, may help to explain the distinct functional effects of A-867744. Thus, our revised structural models may provide a useful tool for interpreting functional effects of PAMs.

## Introduction

Nicotinic acetylcholine receptors (nAChRs) are members of a family of pentameric ligand-gated ion channels (pLGICs) that also includes receptors for 5-hydroxytrptamine, *γ*-aminobutyric acid and glycine (Lester et al., 2004). There has been considerable interest in the identification of positive allosteric modulators (PAMs) of nAChRs (Bertrand and Gopalakrishnan, 2007; Williams et al., 2011; Chatzidaki and Millar, 2015). In particular, extensive efforts have been aimed at the generation of PAMs that are selective for homomeric *α*7 nAChRs (Faghih et al., 2008; Malysz et al., 2009b), a receptor subtype that is implicated in a range of neurological and psychiatric disorders (Haydar and Dunlop, 2010; Parri et al., 2011; Hurst et al., 2013). The *α*7 nAChR undergoes rapid desensitization when activated by the binding of conventional agonists such as acetylcholine to the extracellular orthosteric agonist-binding site (Couturier et al., 1990). However, agonist activation of *α*7 nAChRs is sensitive to modulation by a variety of allosteric ligands (Chatzidaki and Millar, 2015). PAMs acting on *α*7 nAChRs are classified as either type I or type II PAMs, reflecting their differing effects upon agonist-induced desensitization. Whereas type I PAMs have little or no effect on desensitization, type II PAMs cause a reduction in receptor desensitization, as well as potentiating peak agonist responses (Bertrand and Gopalakrishnan, 2007).

**Fig. 1.**
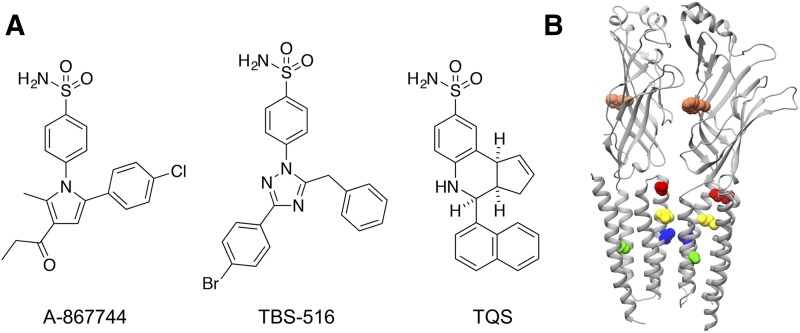
Chemical structures of *α*7 nAChR PAMs and nAChR mutations. (A) The structure of the three compounds examined by electrophysiological techniques are shown (A-867744, TBS-516, and TQS). In addition, a larger group of PAMs with close chemical similarity to these three compounds (7 compounds similar to A-867744, 3 similar to TBS-516, and 24 similar to TQS) were selected for computer docking studies. Details of the chemical structure of all 37 compounds selected for docking studies is provided in Supplemental Table 1. (B) Location of *α*7 nAChR amino acids examined by site-directed mutagenesis. Mutated amino acids are indicated as spheres and correspond to W54 (coral), S222 (green), L247 (blue), M253 (yellow), and M260 (red).

Among the chemically diverse family of *α*7 nAChR PAMs, a subset of compounds share the common feature of an arylsulfonamide group. In the present study, we have examined the pharmacological properties of three arylsulfonamide PAMs [4-(5-(4-chlorophenyl)-2-methyl-3-propionyl-1*H*-pyrrol-1-yl)benzenesulfonamide (A-867744), 4-(5-benzyl-3-(4-bromophenyl)-1*H*-1,2,4-triazol-1-yl)benzene sulfonamide (TBS-516), and *cis*-*cis*-4-(napthalen-1-yl)-3*a*,4,5,9*b*-tetrahydro-3*H*-cyclopenta[*c*]quinoline-8-sulfonamide (TQS)] (Fig. 1) that are representative of three distinct subfamilies of *α*7-selective type II PAMs. A-867744 is a member of a family of PAMs that contains a central pyrrole core (Faghih et al., 2009; Malysz et al., 2009a). TBS-516 is representative of a group of PAMs containing a central triazole core (Chatzidaki et al., 2015), whereas TQS is one of a group of PAMs that contain a cyclopenta[*c*]quinoline core (Grønlien et al., 2007; Gill et al., 2012; Gill-Thind et al., 2015). In common with other *α*7-selective type II PAMs the three compounds potentiate agonist-evoked responses and also reduce desensitization (Grønlien et al., 2007; Malysz et al., 2009a; Gill et al., 2011; Chatzidaki et al., 2015).

For all three families of arylsulfonamide PAMs examined in this study, there is evidence derived from artificial subunit chimeras that the compounds exert their functional effects by binding at a transmembrane site (Malysz et al., 2009a; Gill et al., 2011; Chatzidaki et al., 2015). In addition, competition radioligand binding studies have shown that none of these PAMs displace the binding of antagonists from the *α*7 nAChR orthosteric binding site (Malysz et al., 2009a; Chatzidaki et al., 2015; Gill-Thind et al., 2015). These findings are consistent with the PAMs interacting with a transmembrane allosteric site, as has been proposed for a broader group of *α*7 nAChR allosteric modulators (Young et al., 2008; Collins et al., 2011; Gill-Thind et al., 2015). However, some unexpected results have been reported for A-867744 that are not observed with other *α*7-selective PAMs. Although A-867744 does not displace the binding of the orthosteric antagonist [^3^H]-methyllycaconitine (MLA), it has been reported to displace the binding of an *α*7-selective agonist [(1*S*,4*S*)-2,2-dimethyl-5-(6-phenylpyridazin-3-yl)-5-aza-2-azoniabicyclo[2.2.1]heptane ([^3^H]-A-585539)] that is believed to interact with the orthosteric site (Anderson et al., 2008). This is not necessarily incompatible with A-867744 binding to a transmembrane site, because there is evidence that PAMs interacting with a transmembrane site can induce conformational changes in the orthosteric ligand-binding domain (Barron et al., 2009). Nevertheless, it suggests that A-867744 may cause conformational changes in the receptor that are different from those caused by other PAMs.

In summary, the pharmacological effects of A-867744 have been compared with those of two chemically similar PAMs (TBS-516 and TQS) on both wild-type and mutated *α*7 nAChRs. In addition, two revised comparative models of the human *α*7 nAChRs have been generated based on the closed and open structures of the *Torpedo* nAChR (Unwin and Fujiyoshi, 2012). Importantly, the models were generated after correcting an apparent error in the assignment of amino acids in the transmembrane domain that has been identified previously on the basis of comparisons with other pLGIC structures and biochemical studies (Corringer et al., 2010; Hibbs and Gouaux, 2011; Mnatsakanyan and Jansen, 2013). Molecular docking studies with the revised *α*7 structural models help to explain the pharmacological diversity observed among a group of type II PAMs with close chemically similarity.

## Materials and Methods

### 

#### Chemical Synthesis.

TBS-516 and TQS were synthesized as described previously (Gill et al., 2012; Chatzidaki et al., 2015). A-867744 was purchased from Tocris Bioscience (Bristol, UK).

#### Site-Directed Mutagenesis and Complementary RNA Synthesis.

Site-directed mutagenesis was performed on human nAChR *α*7 subunit cDNA in plasmid pSP64GL (Broadbent et al., 2006) using the QuikChange Mutagenesis Kit (Stratagene, La Jolla, CA) and was verified by nucleotide sequencing. Plasmid pSP64GL containing wild-type or mutated human *α*7 cDNA was linearized with BamHI and purified with QIAQuik PCR Purification Kit (Qiagen, Crawley, UK). In vitro synthesis of complementary RNA was performed using an mMessage mMachine SP6 Transcription Kit (Invitrogen Life Technologies, Carlsbad, CA). For consistency with previous studies, the numbering of amino acids altered by site-directed mutagenesis is based on the predicted signal sequence cleavage site in the chicken *α*7 protein, as reported previously (Couturier et al., 1990).

#### *Xenopus* Oocyte Electrophysiology.

Heterologous expression was achieved by injection of wild-type or mutated *α*7 complementary RNA (6–12 ng) into the cytoplasm of defolliculated *Xenopus laevis* oocytes, as described previously (Young et al., 2007). After injection, oocytes were incubated at 18°C in a calcium-containing Barth’s solution supplemented with antibiotics (100 U/ml penicillin, 100 *μ*g/ml streptomycin, 4 *μ*g/ml kanamycin, and 50 *μ*g/ml tetracycline). Experiments were performed on oocytes after 3–5 days of incubation. Oocytes were placed in a recording chamber and continuously perfused with a saline solution with a flow rate of approximately 15 ml/min. Two-electrode voltage-clamp recordings were performed as described previously (Young et al., 2007; Gill et al., 2012).

#### Radioligand Binding.

Human kidney tsA201 cells were cultured in Dulbecco’s modified Eagle’s medium (Invitrogen Life Technologies) containing 10% fetal calf serum (Sigma-Aldrich, St. Louis, MO), penicillin (100 U/ml), and streptomycin (100 *μ*g/ml) (Invitrogen Life Technologies). Cells were maintained in a humidified incubator containing 5% CO_2_ at 37°C and transiently transfected with expression plasmids (pRK5-h*α*7 pRK5-hRIC-3) encoding human *α*7 nAChR and human RIC-3 (Lansdell et al., 2005) using Effectene (Qiagen), according to the manufacturer instructions. After overnight incubation in Effectene, cells were incubated at 37°C for 24–48 h before being assayed for radioligand binding with [^3^H]-*α*-bungarotoxin ([^3^H]-*α*-BTX) (56 Ci/mmol; Tocris Bioscience). Radioligand binding to transiently transfected tsA201 cells was performed essentially as described previously (Cooper and Millar, 1998; Lansdell and Millar, 2004). Transfected cells were resuspended in Hank’s balanced salt solution (Gibco, Paisley, UK) containing 1% bovine serum albumin and incubated with [^3^H]-*α*-BTX for 2 h at 22°C in a total volume of 150 *μ*l. Nonspecific binding was determined in the presence of nicotine (1 mM) and carbamylcholine (1 mM). Competition binding experiments were performed by incubating triplicate samples of transfected cells with [^3^H]-*α*-BTX (1 nM), together with a range of concentrations of either PAMs or MLA. Radioligand binding was assayed by filtration onto Whatman GF/A filters (presoaked in 0.5% polyethylenimine), followed by rapid washing with phosphate-buffered saline (Oxoid, Basingstoke, UK) using a Brandel (Gaithersburg, MD) cell harvester. Bound radioligand was quantified by scintillation counting.

#### Hierarchical Refinement of *Torpedo* nAChR.

An apparent error in the assignment of amino acids to the 4.0 Å electron cryo-microscopy (cryo-EM) *Torpedo* nAChR density map within the second and third transmembrane (TM) helices (TM2 and TM3) has been identified previously (Corringer et al., 2010; Hibbs and Gouaux, 2011; Mnatsakanyan and Jansen, 2013). Initially, the *Torpedo* *α_γ_* subunit was adjusted with MODELLER version 9.10 (Sali and Blundell, 1993) using a sequence-structure alignment with a shift correction. One hundred models of the *Torpedo* *α_γ_* subunit were built, based on an alignment to the 4 Å atomic model [Protein Data Bank identifyer (PDB ID) 2BG9] (Unwin, 2005) containing a four-residue gap added after the previously assigned position of P236. The intracellular TM3-TM4 helix was removed because it was modeled poorly relative to the rest of the model (due to lack of restraints in this region), and the sequences were realigned at the start of TM4. The model with the highest discrete optimized protein energy (Shen and Sali, 2006) score was then refined via a hierarchical approach in the cryo-EM density maps of the *Torpedo* nAChR in both closed and open conformations at 6.2 Å (Electron Microscopy Data Bank codes EMD-2071 and EMD-2072) (Unwin and Fujiyoshi, 2012) (Supplemental Fig. 1). Initially, each of the maps was segmented around the associated PDB atomic models (PDB IDs 4AQ9 and 4AQ5) using the UCSF Chimera (Resource for Biocomputing, Visualization, and Informatics, University of California, San Francisco, San Francisco, CA) *zone* tool (Pettersen et al., 2004). Densities corresponding to the neighboring subunits and the intracellular TM3-TM4 helix were removed manually. In the first stage of refinement, rigid bodies corresponding to secondary structure elements were determined using the RIBFIND server (Pandurangan and Topf, 2012), and Flex-EM refinement was applied (Topf et al., 2008). In the second stage, to improve the fit of the loop between TM1 and TM2, loop modeling was carried out with MODELLER, generating 200 loops. The loops were ranked by their segment-based cross-correlation coefficient using TEMPy (Farabella et al., 2015), with the highest-ranking loop (with a segment-based cross-correlation coefficient of 0.82) clearly following the density shape. In the third stage, the model containing the refined loop was used as input for normal mode-based flexible fitting with iMODFIT (Lopéz-Blanco and Chacon, 2013) into the density maps of both closed and open conformations of the *Torpedo* nAChR (EMD-2071 and EMD-2072, respectively) (Unwin and Fujiyoshi, 2012). In the final stage, the open and closed models produced by iMODFIT were energy minimized using all-atom conjugate gradient refinement in Flex-EM. For the purpose of this study, we assessed the transmembrane region of the final model using two methods. Agreement with the density was tested using the segment-based Manders’ overlap coefficient (Joseph et al., 2016), and the quality of the model relative to the initial model was assessed by the statistical potential metric ΔQMEANBrane (Studer et al., 2014). PDB files are available for the refined structural models of the *Torpedo* *α_γ_* subunit in the closed and open conformations (Supplemental Material).

#### Revised *α*7 Comparative Models.

Comparative models of the human *α*7 nAChR were generated. The top-ranked closed and open refined models of the *Torpedo* *α_γ_* subunit were used as the templates on which we modeled the human *α*7 nAChR closed and open subunits (Supplemental Fig. 2). One hundred models of *α*7 were generated for each template using MODELLER version 9.10 (Sali and Blundell, 1993). The mean of QMEANBrane scores (calculated over all individual residues) were determined for each model and used as a ranking criterion. The highest-scoring model was selected for each of the closed and open conformations. The corresponding *α*7 subunit models were then superposed on their corresponding templates, and 5-fold symmetry was imposed using the Chimera *sym* command (based on the origin of the closed and open density maps). This created pentameric models for each conformation of *α*7, which were examined using MolProbity (Chen et al., 2010). Suggested side chain flips were applied to reduce clashes, while keeping side chain rotamers as close to the corrected torpedo model as possible. PDB files are available for the human pentameric *α*7 nAChR structural model in the closed and open conformations (Supplemental Material).

#### Small-Molecule Docking.

To identify potential binding sites for PAMs in the *α*7 nAChR closed and open comparative models, a consensus small-molecule docking protocol was developed (Supplemental Fig. 3). Docking was performed with a series of 37 compounds that have close chemical similarity to A-867744 (eight compounds), TBS-516 (four compounds), or TQS (25 compounds), all of which are known to display type II PAM or allosteric agonist/PAM activity on *α*7 nAChRs (for details of the selected compounds, see Supplemental Table 1). Initially, rigid docking was performed with models of the human *α*7 nAChR in both closed and open conformations, and the results obtained with two different docking programs that have been shown to produce reliable results (Wang et al., 2016) [GOLD; Cambridge Crystallographic Data Centre, Cambridge, UK (Verdonk et al., 2003) and AutoDock Vina; Molecular Graphics Lab at The Scripps Research Institute, La Jolla, CA (Trott and Olson, 2010)] were compared. With GOLD, the search area was defined such that all receptor residues within a sphere of 18 Å radius from the *γ*-carbon of T277 of the (+) subunit in the closed model were included. With AutoDock Vina, all receptor residues within a cube of 32 Å centered on T277 of the (+) subunit were included. This search area spanned a region covering both the intersubunit and intrasubunit cavities present in the transmembrane domain. The search efficiency was set to the maximum in both software packages, and the ligands were allowed full flexibility. Fifty diverse solutions were generated in GOLD, and the maximum of 20 solutions was enabled in AutoDock Vina (via 20 runs). The root mean square deviation (RMSD) between the GOLD and AutoDock Vina solutions for each ligand was determined using an in-house python script based on the Hungarian algorithm (Allen and Rizzo, 2014). A centroid was defined based on the location of all atoms in the solutions that had an RMSD of 2.0 Å or less between GOLD and AutoDock Vina. From this centroid, a region was determined that included the positions of all the solutions in the subset (a sphere in GOLD and a box in AutoDock Vina). Ten amino acid side chains were selected for full flexibility within the defined docking areas in both cases. Priority was given to making residues flexible that had been mutated in this study, followed by polar residues and, finally, an even distribution of flexible residues with the aim of preventing any bias to particular regions of the selected binding area. All ligands were docked using the same search parameters that were used for docking studies with the rigid receptor (see above). RMSD filtering was carried out as previously, to remove solutions that had an RMSD greater than 2.0 Å between any of the GOLD and AutoDock solutions. The remaining solutions after redundancy removal (up to 20 pairs) were ranked based on the Borda score (Farabella et al., 2015) using the following three metrics: the AutoDock Vina scoring function, and two scoring functions used in GOLD (GoldScore and ChemPLP; Cambridge Crystallographic Data Centre). After the docking simulation of all ligands, the top five ranked solutions from each chemical class were clustered against each other (based on the GOLD pose) by hierarchical RMSD clustering using an in-house script. The latter step was performed to identify binding modes predicted consistently across multiple compounds with a similar chemical structure. For each of the three classes of arylsulfonamide type II PAMs, the most highly populated clusters were assumed to represent the most probable location of the binding site. PDB files are available for A-867744, TBS-516, and TQS docked into the human *α*7 nAChR structural model in the closed and open conformations (Supplemental Material)

## Results

### 

#### Radioligand Binding.

**Fig. 2.**
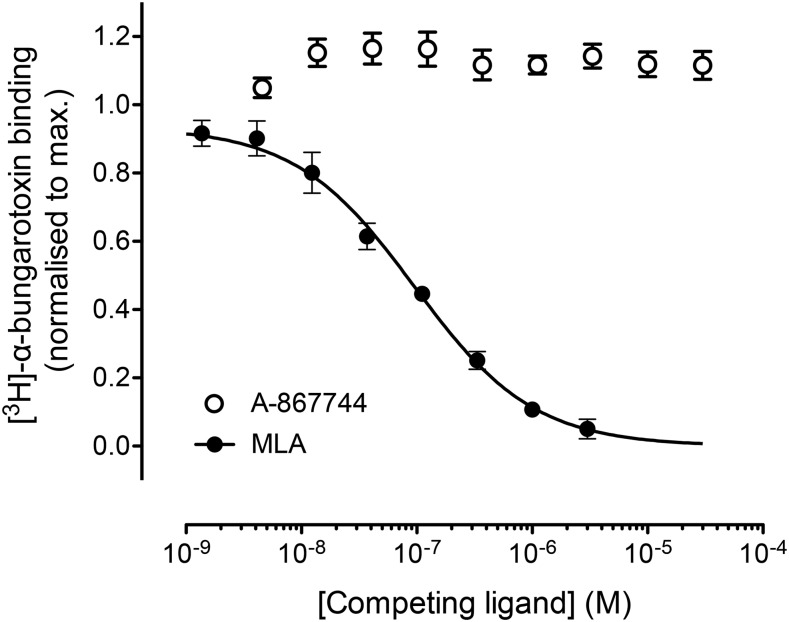
Competition radioligand binding. Equilibrium radioligand binding was performed with [^3^H]-*α*-BTX (1 nM) in human kidney tsA201 cells expressing *α*7 nAChRs. A-867744 (3 nM to 30 *μ*M) caused no significant displacement of [^3^H]-*α*-BTX binding, whereas the orthosteric antagonist MLA caused complete displacement of specific radioligand binding. Data points are means of triplicate samples (±S.E.M.) from a single experiment, and data are typical of three independent experiments.

As has been discussed previously (Malysz et al., 2009a), an unexpected finding concerning the *α*7-selective PAM A-867744 (Fig. 1) is that it causes the dissociation of a radiolabeled agonist ([^3^H]-A-585539) that is believed to bind to the *α*7 extracellular orthosteric binding site. In the present study, we have examined the ability of A-867744 to displace a radiolabeled antagonist ([^3^H]-*α*-BTX) that is known to bind to the *α*7 orthosteric site. A-867744 caused no significant displacement of the binding of [^3^H]-*α*-BTX to *α*7 nAChRs (Fig. 2). In contrast, and as expected, the orthosteric antagonist MLA caused complete displacement of [^3^H]-*α*-BTX (Fig. 2). These findings are consistent with previous studies demonstrating that A-867744 does not displace [^3^H]-MLA from *α*7 nAChRs (Malysz et al., 2009a) and provide additional support for the conclusion that A-867744 does not bind to the orthosteric site.

#### Functional Characterization of *α*7-Selective PAMs.

Given the evidence that A-867744 has properties that are different from those of other *α*7-selective type II PAMs (Malysz et al., 2009a), the functional effects of A-867744, TBS-516, and TQS were examined on *α*7 nAChRs containing five different point mutations. The five mutations examined have all been shown previously to influence allosteric modulation of *α*7 nAChRs (Young et al., 2008; Papke et al., 2014). Three of the mutations are located within TM2 (L247T, M253L, and M260L), one mutation within TM1 (S222M), and one mutation in the extracellular domain, at the orthosteric binding site (W54A). The effects of the five mutations on A-867744, TBS-516, and TQS are summarized in Table 1 and are also described in more detail below.

**TABLE 1:** Pharmacological effects of three arylsulfonamide compounds (A-867744, TBS-516, and TQS) on wild-type *α*7 nAChRs and on *α*7 nAChRs containing single-point mutations

	Wild Type	W54A	S222M	L247T	M253L	M260L
A-867744	PAM	PAM	Inhibitor/PAM*^a^*	Inhibitor	Inhibitor	PAM
TBS-516	PAM	Agonist	PAM	Agonist	<PAM*^b^*	Agonist
TQS	PAM	Agonist	PAM	Agonist	<PAM*^b^*	Agonist

^a^ Positive modulatory effects (reduction in agonist-evoked desensitization) were associated with a reduction in peak agonist-evoked responses.

^b^ Indicates a substantial reduction or abolition of PAM activity.

#### Allosteric Modulation of *α*7^M260L^.

**Fig. 3.**
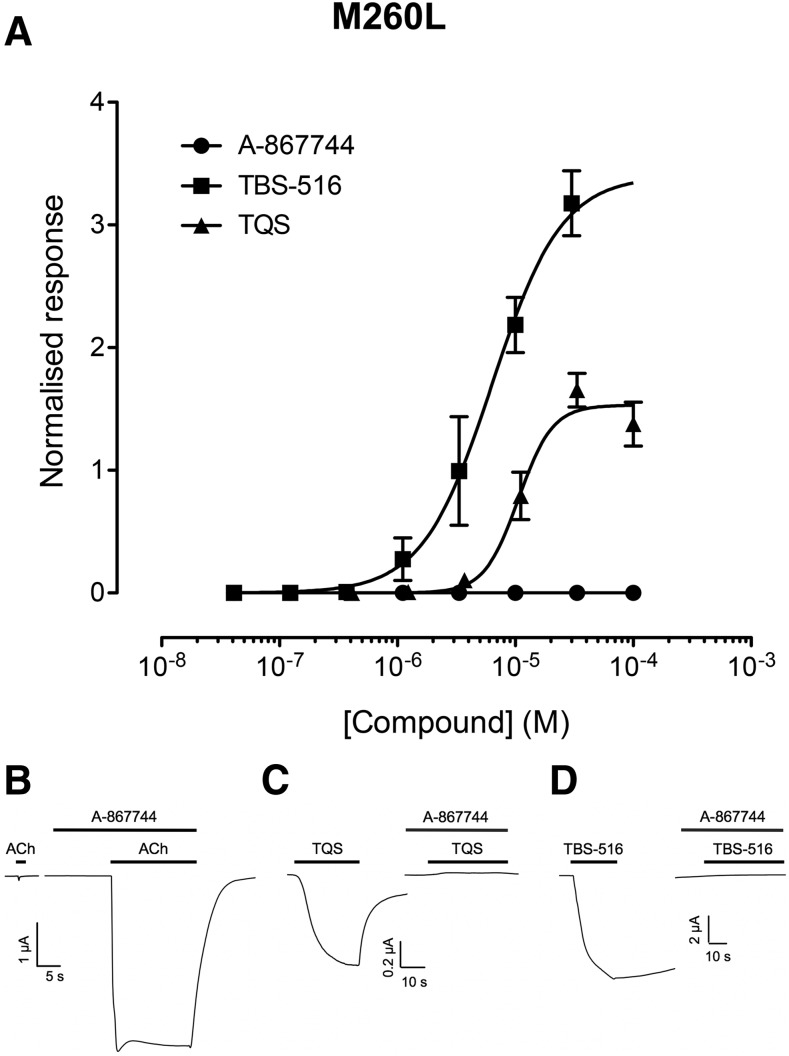
Allosteric modulation of *α*7^M260L^ by A-867744, TBS-516, and TQS. Mutated *α*7^M260L^ nAChRs were expressed in *Xenopus* oocytes and examined by two-electrode voltage-clamp recording. (A) Concentration-response data illustrating agonist activation by TBS-516 and TQS but the absence of agonist activity with A-867744. Data are the mean ± S.E.M. of three independent experiments, each from different oocytes. Data are normalized to the maximum acetylcholine response. (B) Representative traces illustrating responses to acetylcholine (100 *μ*M; left) together with acetylcholine responses from the same oocyte after preapplication (10 seconds) and coapplication of A-867744 (1 *μ*M; right). (C) Representative traces illustrating responses to TQS (30 *μ*M; left) together with TQS responses from the same oocyte after preapplication (10 seconds) and coapplication of A-867744 (1 *μ*M; right). (D) Representative traces illustrating responses to TBS-516 (10 *μ*M; left) together with TBS-516 responses from the same oocyte after preapplication (10 seconds) and coapplication of A-867744 (1 *μ*M; right).

We have reported previously that the M260L mutation, located within the *α*7 nAChR TM2 domain, is able to convert type II PAMs that have little or no agonist effects on wild-type *α*7 nAChRs in allosteric agonists (Chatzidaki et al., 2015). In agreement with these previous findings, TBS-516 and TQS were found to activate *α*7^M260L^ with maximum responses that were higher than acetylcholine (3.7 ± 0.6-fold and 1.5 ± 0.1-fold, respectively) with EC_50_ values of 8.9 ± 2.5 and 12.0 ± 1.1 *μ*M, respectively (Fig. 3A). In marked contrast, A-867744 failed to evoke any agonist responses on *α*7^M260L^ (Fig. 3A), even at high concentrations (100 *μ*M) but, instead, retained its PAM activity (Fig. 3B). Indeed, the potentiation of responses to acetylcholine (100 *μ*M) by A-867744 (1 *μ*M) on *α*7^M260L^ was not significantly different from that on wild-type *α*7 (14.9 ± 5.1-fold and 17.1 ± 2.7-fold, respectively). In addition, preapplication of A-867744 completely blocked the agonist responses elicited by either TBS-516 or TQS on *α*7^M260L^ (Fig. 3, C and D).

#### Allosteric Modulation of *α*7^L247T^.

**Fig. 4.**
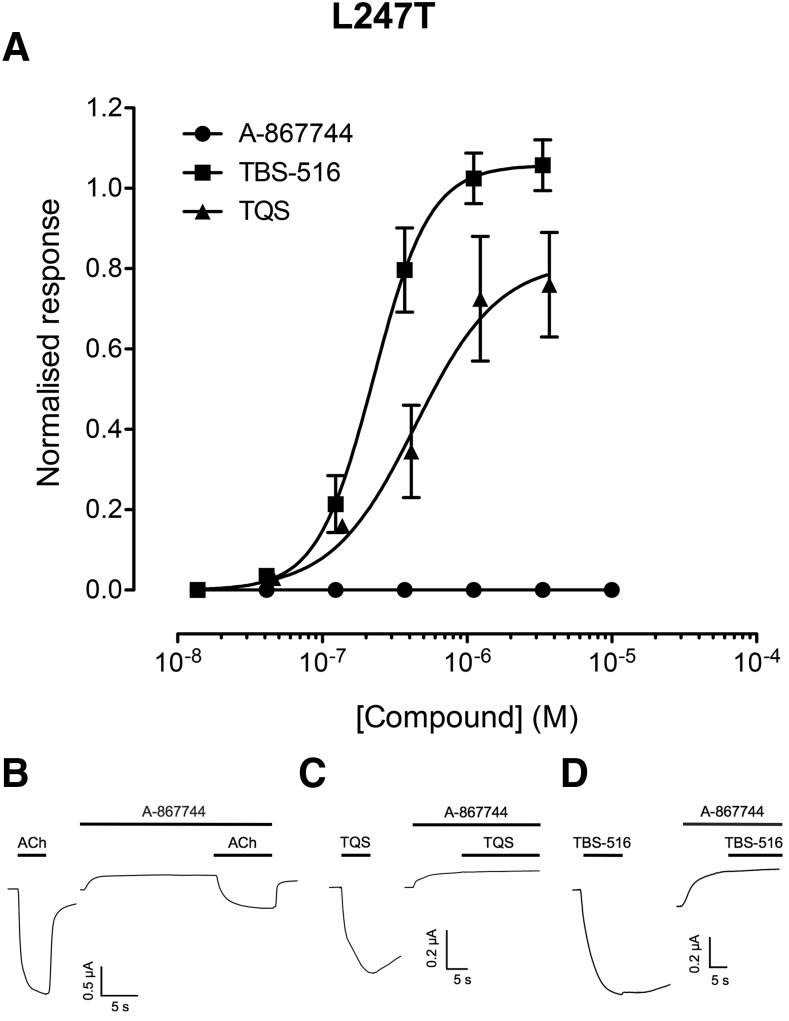
Allosteric modulation of *α*7^L247T^ by A-867744, TBS-516, and TQS. Mutated *α*7^L247T^ nAChRs were expressed in *Xenopus* oocytes and examined by two-electrode voltage-clamp recording. (A) Concentration-response data illustrating agonist activation by TBS-516 and TQS but the absence of agonist activity with A-867744. Data are the mean ± S.E.M. of three independent experiments, each from different oocytes. Data are normalized to the maximum acetylcholine response. (B) Representative traces illustrating responses to acetylcholine (10 *μ*M; left) together with acetylcholine responses from the same oocyte after preapplication (10 seconds) and coapplication of A-867744 (1 *μ*M; right). (C) Representative traces illustrating responses to TQS (10 *μ*M; left) together with TQS responses from the same oocyte after preapplication (10 seconds) and coapplication of A-867744 (1 *μ*M; right). (D) Representative traces illustrating responses to TBS-516 (10 *μ*M; left) together with TBS-516 responses from the same oocyte after preapplication (10 seconds) and coapplication of A-867744 (1 *μ*M; right).

Previous studies have described the effects of the L247T mutation in the *α*7 nAChR (*α*7^L247T^) (Revah et al., 1991). One of the most dramatic effects of this mutation, which is located in the TM2 helix, is to convert a variety of ligands that do not have agonist activity on wild-type nAChRs into agonists. For example, this effect on *α*7^L247T^ has been reported for antagonists (Bertrand et al., 1992; Palma et al., 1996), type I PAMs (Chatzidaki et al., 2015), type II PAMs (Gill et al., 2011), and also for silent allosteric modulators (Gill-Thind et al., 2015). In agreement with these previous studies, we have found that both TBS-516 and TQS act as agonists on *α*7^L247T^ with EC_50_ values of 0.4 ± 0.1 and 0.2 ± 0.04 *μ*M, respectively (Fig. 4A). In contrast, no agonist activity was observed on *α*7^L247T^ with A-867744 (Fig. 4A). Whereas A-867744 acts as a PAM when coapplied with acetylcholine on wild-type *α*7 nAChRs (Malysz et al., 2009a), the coapplication of A-867744 (1 *μ*M) on *α*7^L247T^ inhibited responses to a maximal concentration of acetylcholine (10 *μ*M) (66% ± 1.2%) (Fig. 4B). In addition, the same concentration of A-867744 completely blocked agonist responses to a maximal concentration of TBS-516 (10 *μ*M) and TQS (10 *μ*M) (Fig. 4, C and D). It is noteworthy that the application of A-867744 alone caused a decrease in the baseline current on *α*7^L247T^ (Fig. 4B). Previous studies have reported that the L247T mutation causes an increase in the frequency of spontaneous openings in *α*7 nAChRs (Bertrand et al., 1997), so it is likely that A-867744 may be inhibiting these spontaneous openings in *α*7^L247T^.

#### Allosteric Modulation of *α*7^M253L^.

**Fig. 5.**
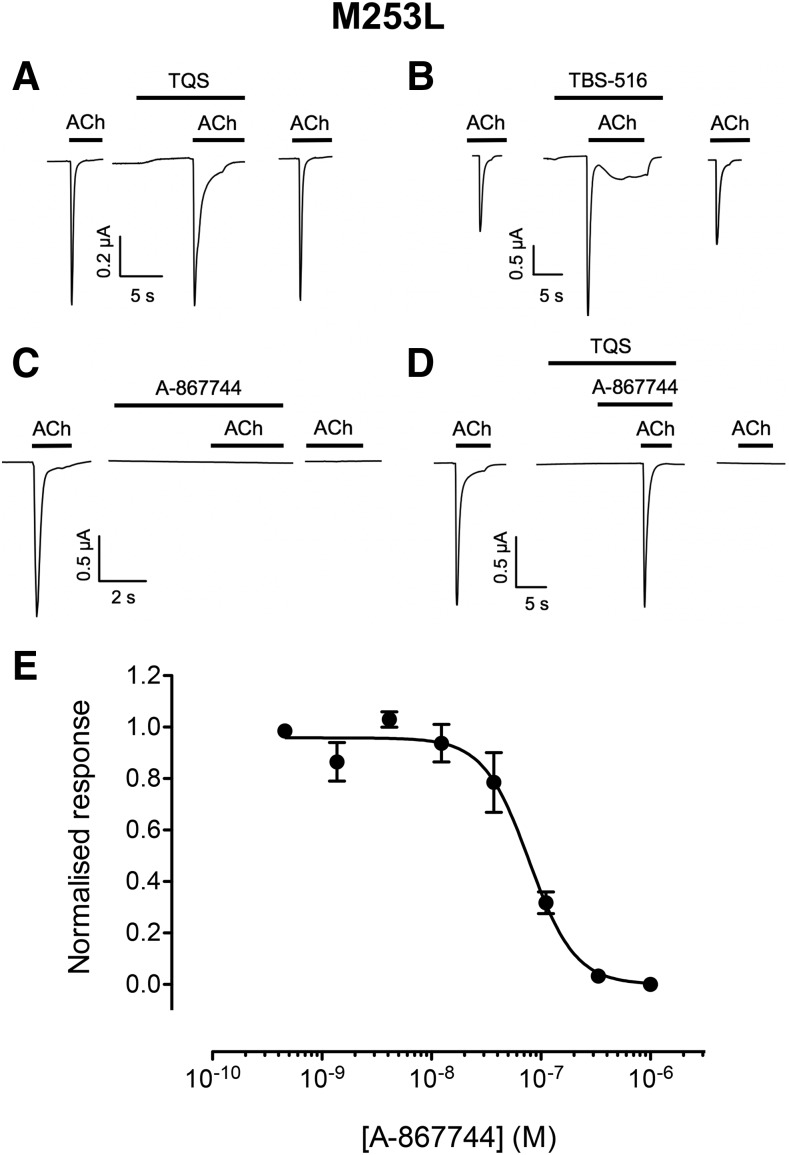
Allosteric modulation of *α*7^M253L^ by A-867744, TBS-516, and TQS. Mutated *α*7^M253L^ nAChRs were expressed in *Xenopus* oocytes and examined by two-electrode voltage-clamp recording. (A) Representative traces illustrating responses to acetylcholine (100 *μ*M; left), acetylcholine responses after preapplication (10 seconds) and coapplication of TQS (10 *μ*M; middle), and response to acetylcholine after washing (right). (B) Representative traces illustrating responses to acetylcholine (100 *μ*M; left), acetylcholine responses after preapplication (10 seconds) and coapplication of TBS-516 (10 *μ*M; middle), and response to acetylcholine after washing (right). (C) Representative traces illustrating responses to acetylcholine (100 *μ*M; left), and block of acetylcholine responses after preapplication (10 seconds) and coapplication of A-867744 (1 *μ*M; middle). The acetylcholine response did not recover, even after a prolonged (10 minutes) wash (right). (D) Representative traces illustrating responses to acetylcholine (100 *μ*M; left) and after preapplication of TQS (10 seconds), preapplication of A-867744 and TQS (5 seconds), and then coapplication of A-867744 (1 *μ*M) and TQS (10 *μ*M) (middle). TQS was preapplied for 10 seconds and A-867744 for 5 seconds. The acetylcholine response did not recover, even after a prolonged (10 minutes) wash (right). (E) Concentration-response data illustrating inhibition by A-867744 of responses to acetylcholine (100 *μ*M). Data are the mean ± S.E.M. of four independent experiments, each from different oocytes.

The influence of a third mutation (M253L) within the TM2 domain of *α*7 (*α*7^M253L^) was examined. M253L has been reported previously to reduce or abolish the potentiating effect of several allosteric ligands (Young et al., 2008; Gill et al., 2011). In agreement with these previous studies, coapplication of TQS (10 *μ*M) with acetylcholine (100 *μ*M) on *α*7^M253L^ did not result in an increase in peak current (Fig. 5A). Furthermore, coapplication of TBS-516 (10 *μ*M) with acetylcholine (100 *μ*M) on *α*7^M253L^ resulted in only a small increase in peak current (1.8 ± 0.2-fold potentiation; *n* = 4) (Fig. 5B). In contrast, A-867744 (1 *μ*M) acted as an antagonist, producing a complete block of responses to acetylcholine (100 *μ*M) with an IC_50_ value of 70.2 ± 13.5 nM (Fig. 5, C and E). The block by A-867744 was surprisingly long lasting, as illustrated by responses to acetylcholine failing to recover even after a long (10 minutes) wash (Fig. 5C). Interestingly, when TQS was preapplied before the application of A-867744, antagonism of acetylcholine responses by A-867744 was not observed (Fig. 5D). However, after washing to remove TQS and A-867744, no subsequent response to acetylcholine could be detected, even after a prolonged wash (Fig. 5D). This may be because A-867744 dissociates more slowly from the receptor than TQS or perhaps that A-867744 is able to reassociate with the receptor during the wash period.

#### Allosteric Modulation of *α*7^S222M^.

**Fig. 6.**
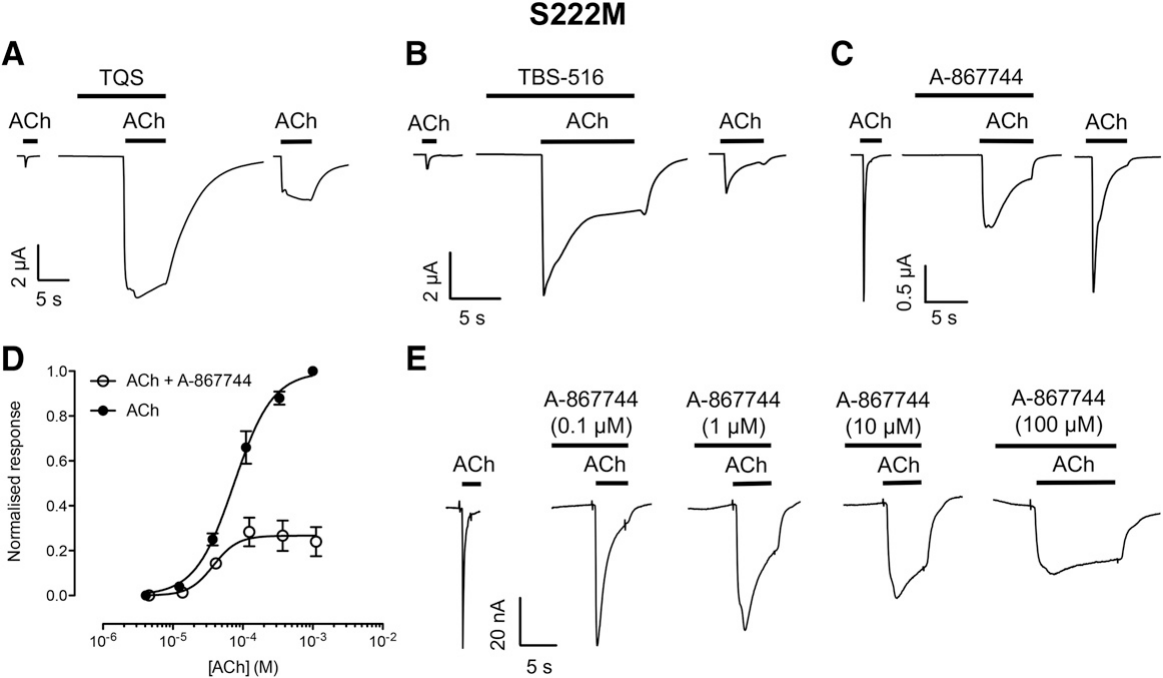
Allosteric modulation of *α*7^S222M^ by A-867744, TBS-516, and TQS. Mutated *α*7^S222M^ nAChRs were expressed in *Xenopus* oocytes and examined by two-electrode voltage-clamp recording. (A) Representative traces illustrating responses to acetylcholine (100 *μ*M; left), acetylcholine responses after preapplication (10 seconds) and coapplication of TQS (10 *μ*M; middle), and response to acetylcholine after washing (right). (B) Representative traces illustrating responses to acetylcholine (100 *μ*M; left), acetylcholine responses after preapplication (10 seconds) and coapplication of TBS-516 (10 *μ*M; middle), and response to acetylcholine after washing (right). (C) Representative traces illustrating responses to acetylcholine (100 *μ*M; left), block of acetylcholine responses after preapplication (10 seconds) and coapplication of A-867744 (1 *μ*M; middle), and response to acetylcholine after washing (right). (D) Agonist concentration-response curve for acetylcholine in the absence and presence of A-867744 (1.5 *μ*M; preapplied for 10 seconds and then coapplied with acetylcholine). Data are the mean ± S.E.M. of at least three independent experiments and are normalized to the maximum response obtained with ACh in the absence of A-867744. (E) When A-867744 was preapplied and coapplied with acetylcholine, positive modulatory effects (a reduction in agonist-evoked desensitization) was associated with a reduction in peak agonist responses.

A mutation (S222M) located in the TM1 domain of *α*7 (*α*7^S222M^) has been reported to reduce but not abolish the potentiation of acetylcholine responses by the type II PAM PNU-120596 (Young et al., 2008). In agreement with these findings, both TBS-516 and TQS retained PAM activity on *α*7^S222M^ by potentiating peak responses and reducing desensitization (Fig. 6, A and B). In contrast, whereas A-867744 also caused a reduction in desensitization, it caused a reduction of agonist-evoked responses on *α*7^S222M^ (Fig. 6C). Studies with a range of A-867744 concentrations indicate an incomplete block, reducing responses to acetylcholine to 33% ± 3.0% (*n* = 7) with IC_50_ value of 3.1 ± 1.4 *μ*M (*n* = 4) (Fig. 6, D and E). Acetylcholine concentration-response curves obtained in the absence and presence of A-867744 (1.5 *μ*M) show a nonsurmountable block by A-867744 (Fig. 6D), which is characteristic of noncompetitive antagonism. The acetylcholine EC_50_ values were not significantly different for the curves in the absence and presence of A-867744 (80.6 ± 16.2 and 37.8 ± 27.2 *μ*M, respectively; *P* = 0.06; *n* = 3). However, maximum current (*I*_max_) in the presence of A-867744 was significantly reduced (26.4% ± 6.6% of control; *P* < 0.001, *n* = 3).

#### Allosteric Modulation of *α*7^W54A^.

**Fig. 7.**
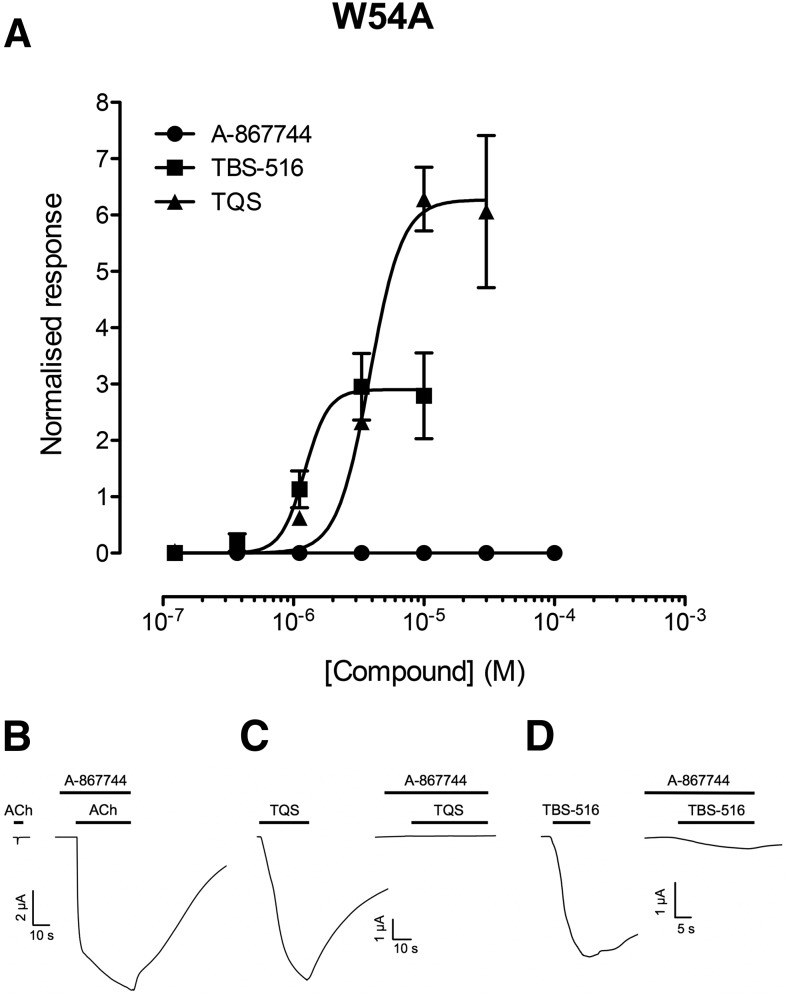
Allosteric modulation of *α*7^W54A^ by A-867744, TBS-516, and TQS. Mutated *α*7^W54A^ nAChRs were expressed in *Xenopus* oocytes and examined by two-electrode voltage-clamp recording. (A) Concentration-response data illustrating agonist activation by TBS-516 and TQS but the absence of agonist activity with A-867744. Data are the mean ± S.E.M. of three independent experiments, each from different oocytes. Data are normalized to the maximum acetylcholine response. (B) Representative traces illustrating responses to acetylcholine (100 *μ*M; left) together with acetylcholine responses from the same oocyte after preapplication (10 seconds) and coapplication of A-867744 (1 *μ*M; right). (C) Representative traces illustrating responses to TQS (10 *μ*M; left) together with TQS responses from the same oocyte after preapplication (10 seconds) and coapplication of A-867744 (1 *μ*M; right). (D) Representative traces illustrating responses to TBS-516 (10 *μ*M; left) together with TBS-516 responses from the same oocyte after preapplication (10 seconds) and coapplication of A-867744 (1 *μ*M; right).

In addition to transmembrane mutations influencing allosteric modulation of *α*7 nAChRs, dramatic effects have also been reported for a mutation (W54A) located in the extracellular domain, near the orthosteric binding site. For example, it has been reported that type II PAMs are converted into agonists in *α*7^W54A^ (Papke et al., 2014). In agreement with these previous findings, both TBS-516 and TQS exhibited agonist effects on *α*7^W54A^ with EC_50_ values of 4.1 ± 0.6 and 1.5 ± 0.5 *μ*M, respectively (Fig. 7A). In contrast, A-867744 had no detectable agonist activity on *α*7^W54A^ but retained its PAM activity (Fig. 7B). The maximum fold potentiation of the response to acetylcholine (100 *μ*M) by A-867744 was 15.1 ± 1.8-fold (*n* = 12), which is not significantly different from that observed with wild-type *α*7 nAChRs (17.1 ± 2.7-fold; *n* = 7; *P* = 0.54). In addition, it was found that A-867744 (1 *μ*M) blocked agonist responses elicited by TBS-516 and TQS on *α*7^W54A^ (Fig. 7, C and D).

#### Refined *α*7 nAChR Comparative Models.

**Fig. 8.**
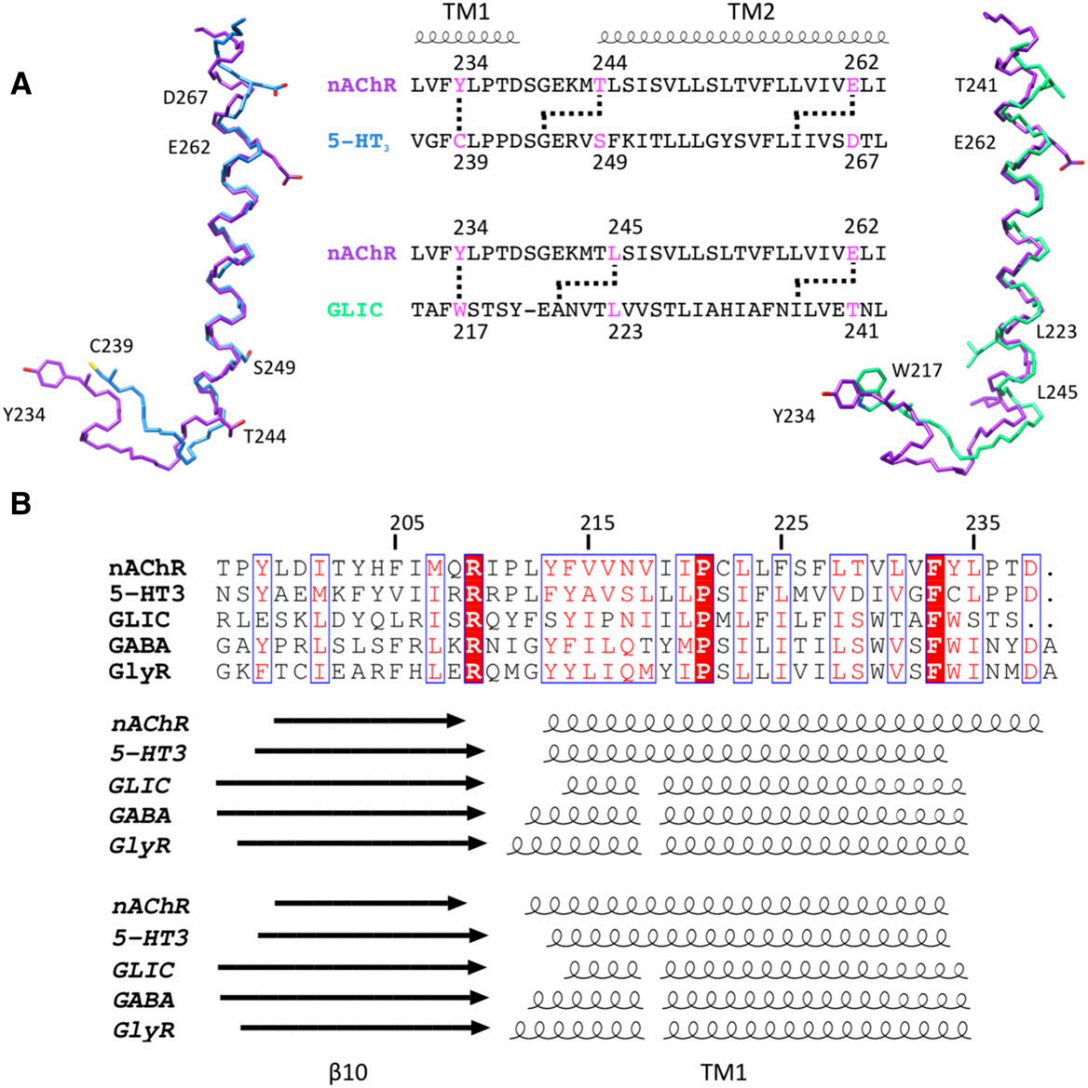
Refinement of the transmembrane domain of the *Torpedo* nAChR *α_γ_* subunit. (A) Sequence alignment and structural superposition of the TM1-TM2 loop region and TM2 helix of the *Torpedo* *α_γ_* subunit (PDB ID 2BG9; chain A) with that of the 5-HT_3_ receptor (PDB ID 4PIR; chain A) and GLIC (PDB ID 4HFI; chain A). As has been described previously (Supplemental Fig. 21 in Hibbs and Gouaux, 2011), amino acids from the TM1-TM2 loop of the *Torpedo* nAChR (e.g., Y234) superpose well with homologous amino acids from other pLGIC structures (C239 of 5-HT_3_R and W217 of GLIC; indicated by a straight line in the sequence alignment). In contrast, amino acids within the nAChR TM2 domain are out of register by ∼1 turn of the *α*-helix when compared with other pLGIC structures (e.g., compare E262 of the nAChR structure with D267 of 5-HT_3_R and T241 of GLIC; indicated by an angled line in the sequence alignment). (B) Alignment of amino acid sequence of the *β*10 strand and TM1 helix of *Torpedo* nAChR *α* subunit with that of related pLGICs (top panel). Also shown is the secondary structure before refinement (middle panel) and after refinement (bottom panel) of the *Torpedo* nAChR structure. Structural information is derived from the following PDB files: nAChR (2BG9; chain A), 5-HT_3_ receptor (4PIR; chain A), GABA_A_ receptor (4COF; chain A), glycine receptor (3JAD; chain A), and GLIC (4HFI; chain A). Arrows denote *β*-strands, spirals denote *α*-helices, conserved residues are highlighted with white text on a red background, and residues with similar properties are highlighted with red text on a white background.

To examine by computational approaches the possible interaction of PAMs with the *α*7 nAChR, comparative structural models were constructed based on the cryo-EM structures of the *Torpedo* nAChR in its closed and open states (Unwin, 2005; Unwin and Fujiyoshi, 2012). A previously reported error in the register of amino acids within the transmembrane domain of the 4.0 Å resolution *Torpedo* nAChR structure has been examined. Specifically, it appears that the register of amino acids in TM2 and TM3 is out by four amino acids due to the assignment of an extra turn of the *α*-helix in TM1 (Corringer et al., 2010; Hibbs and Gouaux, 2011; Mnatsakanyan and Jansen, 2013). The discrepancy was identified as beginning after the residue Y234 in TM1 (Fig. 8, A and B). In the present study, this error has been corrected via a hierarchical refinement protocol using additional cryo-EM density maps of *Torpedo* nAChR in closed and open conformations (*Materials and Methods*) (Supplemental Fig. 1; Supplemental Table 2). After correction, the *α*-helical region of TM1 terminates at F233, a position analogous to that of the 5-HT_3_ receptor (Fig. 8B). The corrected closed and open structures of the *Torpedo* nAChR *α_γ_* subunit were used as a template on which to model the human *α*7 nAChR on the basis of their close sequence identity of 46% overall in the transmembrane region and 54% for the TM1-TM3 region (Supplemental Fig. 2; Supplemental Table 2). Models of the *α*7 subunit were generated that contained the extracellular and transmembrane domains but omitted the more divergent intracellular TM3-TM4 loop. Pentameric *α*7 nAChR models were then constructed by applying 5-fold symmetry (*Materials and Methods*) (Supplemental Fig. 3). Having corrected the assignment of amino acid positions within the transmembrane domain of the *Torpedo* nAChR models, the *α*7 comparative models had the same register of amino acids as found in an NMR structure of the *α*7 transmembrane domain (Mowrey et al., 2013). In addition, comparison between the open and closed conformations revealed a concerted movement of all four helices in the TM domain. Analysis of the channel lumen of the two *α*7 structural models with PoreWalker (European Bioinformatics Institute, Wellcome Genome Campus, UK) (Pellegrini-Calace et al., 2009) revealed a dilation between the closed and open *α*7 comparative models (Supplemental Fig. 3).

#### Molecular Docking.

**Fig. 9.**
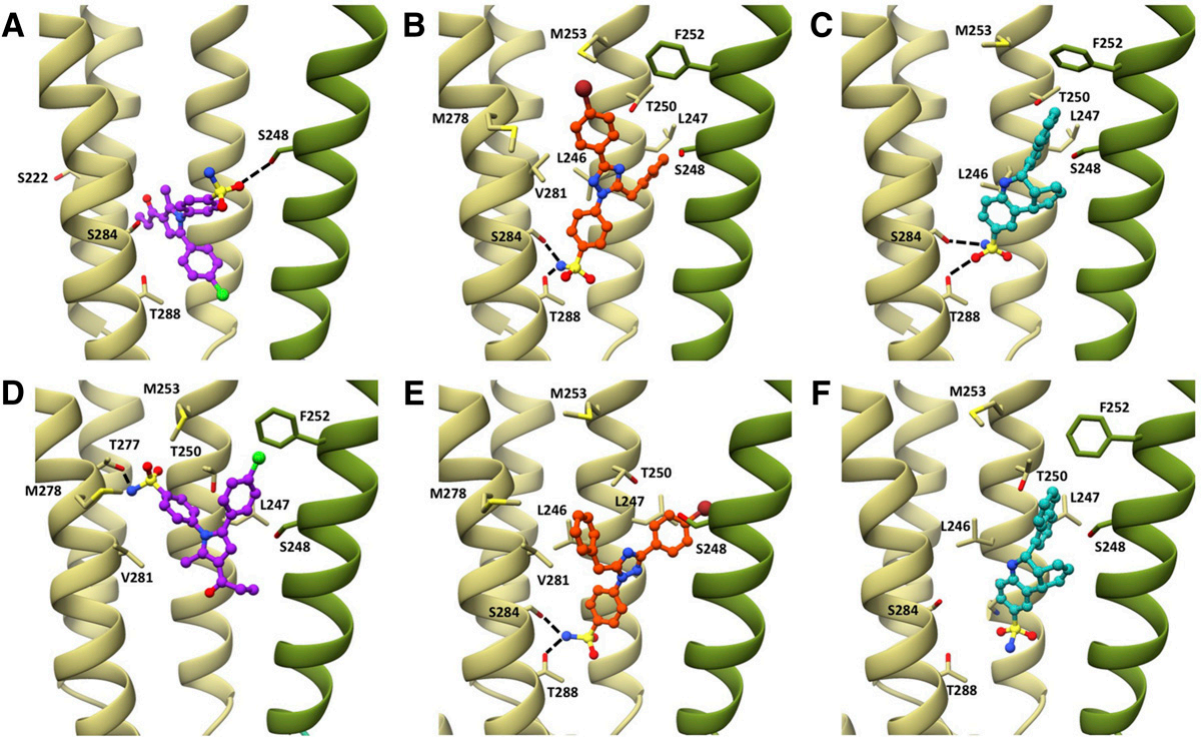
Docking of A-867744, TBS-516, and TQS and into *α*7 structural models. After docking studies with the closed and open models (top and bottom panels, respectively), representatives of binding mode clusters are illustrated for A-867744 (A and D; purple). TBS-516 (B and E; orange), and TQS (C and F; cyan). Amino acids that are discussed in the text are shown in stick representation. Predicted hydrogen bonds are shown with dashed lines. In each case, the principal subunit is shown in khaki (on the left in each panel) and the complimentary subunit is shown in olive (on the right in each panel).

To identify potential binding sites for PAMs in the *α*7 nAChR closed and open comparative models, a consensus small-molecule docking protocol was developed (see *Materials and Methods*) (Supplemental Fig. 4). Docking was performed with a series of 37 compounds that have close chemical similarity to A-867744 (eight compounds), TBS-516 (four compounds), or TQS (25 compounds), all of which are known to display type II PAM activity on *α*7 nAChRs (for details of the selected compounds, see Supplemental Table 1). After the docking simulation, the top five ranked solutions from each chemical class were clustered against each other by hierarchical RMSD clustering. The latter step was performed to identify binding modes predicted consistently across multiple compounds with a similar chemical structure (*Materials and Methods*). For each of the three classes of arylsulfonamide type II PAMs, the most highly populated clusters were assumed to represent the most probable location of the binding site. The docking protocol resulted in the largest binding mode clusters located in an intersubunit cavity (Fig. 9). For the group of compounds chemically related to TBS-516 and TQS, a high degree of similarity was observed between the binding mode clusters identified in the closed and open models (Fig. 9). The arylsulfonamide groups were predicted to interact with the lower TM2-TM3 interface, forming hydrogen bonds with S284 and/or T288 (Fig. 9). In contrast, the predicted consensus binding modes for the series of compounds related to A-867744 were in significantly different orientations to the group of PAMs with chemical similarity to TBS-516 and TQS (Fig. 9). For example, in both conformations the predicted consensus binding mode did not show any hydrogen bonding with S284 or T288. Furthermore, the arylsulfonamide group, a common fragment in all three classes of compound, was predicted to form very different interactions for the A-867744 compound set than for the TBS-516 or TQS compound sets (Fig. 9). Interface sizes of the docked protein-ligand complexes were determined using the Protein Interfaces, Surfaces, and Assemblies service PISA at the European Bioinformatics Institute (Krissinel and Henrick, 2007) and were found to be comparable to those in the template structures (Supplemental Table 3).

#### Predicted Binding of TBS-516 and TQS.

Predicted binding mode clusters for both TBS-516 and TQS and their related compounds occupy strikingly similar locations in both closed and open receptor models (Fig. 9; Supplemental Fig. 7). Notably, in the closed conformation, potential hydrogen bonds were observed for the arylsulfonamides of both compounds with the residues S284 and T288 at the cytoplasmic end of TM3 (Fig. 9). These residues are involved in the TM2-TM3 interface, possibly forming part of the desensitization gate of pLGICs (Gielen et al., 2015). Consequently, such interactions may help to explain changes in the rate of desensitization that are observed with these type II PAMs. The naphthyl ring of TQS sits in a hydrophobic pocket formed by the side chains of the amino acids L247, S248, and F252 of the (−) subunit, and L246, L247, T250, and M253 of the (+) subunit, and is situated ∼6.5 Å from M253 and ∼5.0 Å from L247, both of which are amino acids that have been mutated in this study. In the TBS-516 compounds, a similar pocket is occupied by either the benzyl ring (4.0 Å to M253 and 3.5 Å to L247) or the bromophenyl ring (7.2 Å to M253 and 3.5 Å to L247) in the closed and open conformations, respectively. The close proximity of TQS and TBS-516 to M253 and L247 may explain their observed pharmacological effects on the respective mutants.

In the open conformation, the TQS naphthyl ring remains in the same hydrophobic pocket (Fig. 9). Unlike TBS-516, the arylsulfonamide of TQS is farther away from S284 and T288, no longer forming hydrogen bonds with these residues (Fig. 9) but is within ∼3.5 Å of G242, another residue that has been suggested to contribute to the desensitization gate of pLGICs (Gielen et al., 2015). Nonetheless, the cluster of binding modes for TQS predicted in the open conformation covers a broader area than that predicted in the closed conformation (Fig. 9), and many of the other members of the cluster maintain hydrogen bonding interactions with S284 and T288, as well as forming hydrogen bonds to the protein backbone at G242. For TBS-516, the bromophenyl ring sits in the same hydrophobic pocket as the naphthyl ring of TQS (Fig. 9), whereas the third variable group (a benzyl group) protrudes onto a different hydrophobic surface, formed by L246, T250, M253, M278, and V281 (Fig. 9).

#### Predicted Binding of A-867744.

The predicted binding modes of A-867744 and related compounds are in a location similar to those of TBS-516 and TQS but have subtle yet important differences (Fig. 9). Most obviously, whereas the arylsulfonamide group of the TBS-516 and TQS clusters is orientated downward in both the open and closed receptor conformation, it is orientated upward for the A-867744 cluster (Fig. 9; Supplemental Fig. 7) In the closed conformation, the arylsulfonamide group of A-867744 forms a hydrogen bond with S248 of the (−) subunit (an amino acid predicted to interact with both TBS-516 and TQS). However, the orientation of the molecule is different from that of TBS-516 and TQS, situated between the TM2 and TM3 helices, with a protrusion into the intrasubunit cavity, ∼7.0 Å from S222 (another amino acid mutated in this study) (Fig. 9). TBS-516 and TQS are predicted to lie much farther from this residue in the closed conformation (∼14.0 Å), which may explain how the S222M mutation has a dramatic effect on A-867744, but not on TBS-516 or TQS (Fig. 9).

Interestingly, in the open conformation, the predicted binding mode for A-867744 is very different from its closed conformation counterpart (closer to the extracellular domain) (Fig. 9). The arylsulfonamide appears to form hydrogen bonds with T277 and T250, which is markedly different from its interactions in TQS or TBS-516 (Fig. 9); however, it is still located in the TM2-TM3 interface, perhaps influencing the possible desensitization gate. The phenyl ring of the arylsulfonamide is located on the same hydrophobic surface as described for TBS-516 (Fig. 9). The chlorophenyl ring is also predicted to be embedded in the same hydrophobic pocket as that described for the naphthyl ring of TQS, close to L247 and M253 (Fig. 9).

## Discussion

The pharmacological properties of three type II PAMs (A-867744, TBS-516, and TQS) have been examined on five different mutants of the *α*7 nAChR (W54A, S222M, L247T, M253L, and M260L). In all cases, the effects on the action of A-867744 were different from those observed with two other arylsulfonamide type II PAMs (Table 1). This is consistent with previous studies that have suggested that A-867744 is an *α*7 PAM with unusual properties (Malysz et al., 2009a).

Prior to the present study, one of the primary reasons for concluding that A-867744 had atypical properties was that it caused dissociation of [^3^H]-A-585539 (Malysz et al., 2009a), an *α*7-selective agonist that is thought to bind at the orthosteric site (Anderson et al., 2008). However, studies with subunit chimeras suggest that A-867744 exerts its potentiating effects by binding at a transmembrane site (Malysz et al., 2009a). This is also consistent with evidence that A-867744 does not displace binding of orthosteric antagonists such as [^3^H]-*α*-BTX (the present study) or [^3^H]-MLA (Malysz et al., 2009a). One possible explanation is that the binding of A-867744 to a transmembrane binding site results in the stabilization of a different open conformation to that of other type II PAMs.

Numerous *α*7 mutations have been reported to alter the properties of orthosteric and allosteric ligands. Although some mutations, such as M260L, have differing effects on type I and type II PAMs (Chatzidaki et al., 2015), mutations generally appear to have consistent effects on compounds that have similar effects on wild-type *α*7 nAChRs, such as type II PAMs (Chatzidaki et al., 2015). The identification of mutations that have differing effects on A-867744 than those on other type II PAMs, provides evidence that A-867744 has an atypical mechanism of action. An explanation might be that A-867744 binds at an allosteric site on *α*7 that is different from the binding sites of other PAMs. This could be due to the PAMs interacting in different ways at a common or overlapping site, or perhaps binding at unrelated sites. Binding to a common site would be consistent with the observation that A-867744 is able to block the effects of the other PAMs on these mutants.

Recently, an X-ray structure of the *α*4*β*2 nAChR (PDB ID 5KXI) has been determined (Morales-Perez et al., 2016). In addition, the structure of the transmembrane domain of the *α*7 nAChR has been studied by NMR but corresponds to an unassembled subunit monomer and contains deletions and amino acid substitutions (Mowrey et al., 2013). Both of these structures were derived from detergent-solubilized proteins, and, because of concerns that this may influence transmembrane structure, they were not selected as a starting point for the docking studies. Additionally, when an *α*7 model built on an *α*4 subunit of 5KXI was subjected to our docking protocol, no consensus solutions were identified. This provides support for the assumption that crystal structures may be less appropriate starting points for docking to pLGIC transmembrane domains due to the absence of membrane lipids causing tighter packing of the transmembrane domain, as reflected by the increased interface size between subunits of *α*4*β*2 and *Torpedo* nAChR models (Supplemental Table 3).

Our main rationale for using the *Torpedo* nAChR to generate *α*7 models is that it is the only pLGIC structure to have been determined in a lipid membrane environment. This may be important in preserving the native structure of the transmembrane domain. Thus, our approach was to use the *Torpedo* nAChR structure to generate an *α*7 model but also to correct an error that had been identified in the assignment of amino acids within the transmembrane domain of the *Torpedo* nAChR model (Corringer et al., 2010; Hibbs and Gouaux, 2011; Mnatsakanyan and Jansen, 2013). A further benefit of using the *Torpedo* nAChR is that structures were determined in both the closed and open conformations, allowing us to generate two analogous conformations of *α*7.

Evaluation of our corrected structures suggested that they represented a higher-quality model of the transmembrane region (Supplemental Fig. 5). The gating motion between closed and open conformations of our comparative models reveals a concerted rotation of the four TM helix bundle of each domain, which is similar to gating mechanisms that have been proposed from studies with other pLGICs (Calimet et al., 2013; Du et al., 2015).

Ligands binding to transmembrane regions are less likely to form high-affinity interactions due to the lack of polar residues, and this can limit the success of docking studies. Therefore, we used a consensus docking approach that examined a group of compounds with chemical and pharmacological similarity to the three compounds of primary interest (A-867744, TBS-516, and TQS) in two independent docking programs. Our justification being that previous studies have suggested that a consensus docking approach can improve the reliability of docking predictions (Houston and Walkinshaw, 2013).

All three groups of type II PAMs were predicted to bind in the intersubunit transmembrane region (Fig. 9). This is different from what was previously predicted for some allosteric modulators of *α*7 (e.g., LY-2087107, NS-1738, and PNU-120596) where an intrasubunit site was proposed (Young et al., 2008; Collins et al., 2011; Gill et al., 2011). We attribute this difference to the previous studies being conducted with *α*7 comparative models that were based upon an uncorrected model of the *Torpedo* nAChR. Indeed, we have also now performed consensus docking studies with LY-2087107, NS-1738, and PNU-120596 in our new *α*7 comparative model and found that these PAMs are also predicted to bind in an intersubunit site (Supplemental Fig. 6).

Our prediction of an intersubunit binding site for *α*7 allosteric modulators is consistent with the fact that another allosteric modulator (ivermectin) is known to bind at the intersubunit cavity, based on cocrystallization with two different pLGICs (Althoff et al., 2014; Du et al., 2015). In addition, an intersubunit transmembrane binding site has been identified on the basis of affinity-labeling studies with purified *Torpedo* nAChRs and photoreactive allosteric modulators (Nirthanan et al., 2008; Hamouda et al., 2011).

Our experimental data are consistent with A-867744, TBS-516, and TQS binding in a mutually exclusive manner to an overlapping binding site. For example, preapplication of A-867744 to *α*7^L247T^ prevents allosteric activation by TBS-516 and TQS (Fig. 4). Similarly, preapplication of TQS to *α*7 M253L prevents antagonism by A-867744 (Fig. 5). However, our finding that A-867744 exerts pharmacological effects distinct from those of TBS-516 and TQS on mutated *α*7 nAChRs suggests that A-867744 interacts by a different mechanism. It is interesting that a mutation in the extracellular domain (W54A) influences the activity of these PAMs (presumably, a consequence of long-range effects on receptor structure), yet it has a different effect on A-867744 than on TBS-516 and TQS (Fig. 7; Table 1) This is consistent with previous evidence indicating, unexpectedly, that A-867744 can cause the dissociation of [^3^H]-A-585539 from the orthosteric site (Malysz et al., 2009a).

Our docking studies appear to be consistent with the experimental findings. Docking studies suggest that A-867744 binds in an orientation that is distinct from that of both TBS-516 and TQS (most apparent in the closed model) (Fig. 9; Supplemental Fig. 7). This provides a basis for understanding how both proximal and distant mutations affect the function of A-867744 differently from how they affect TBS-516 and TQS. However, despite these differences, all three compounds are predicted to bind in a broadly similar transmembrane location that is in proximity to residues that have been implicated in the desensitization gate of pLGICs (Gielen et al., 2015).

**Fig. 10.**
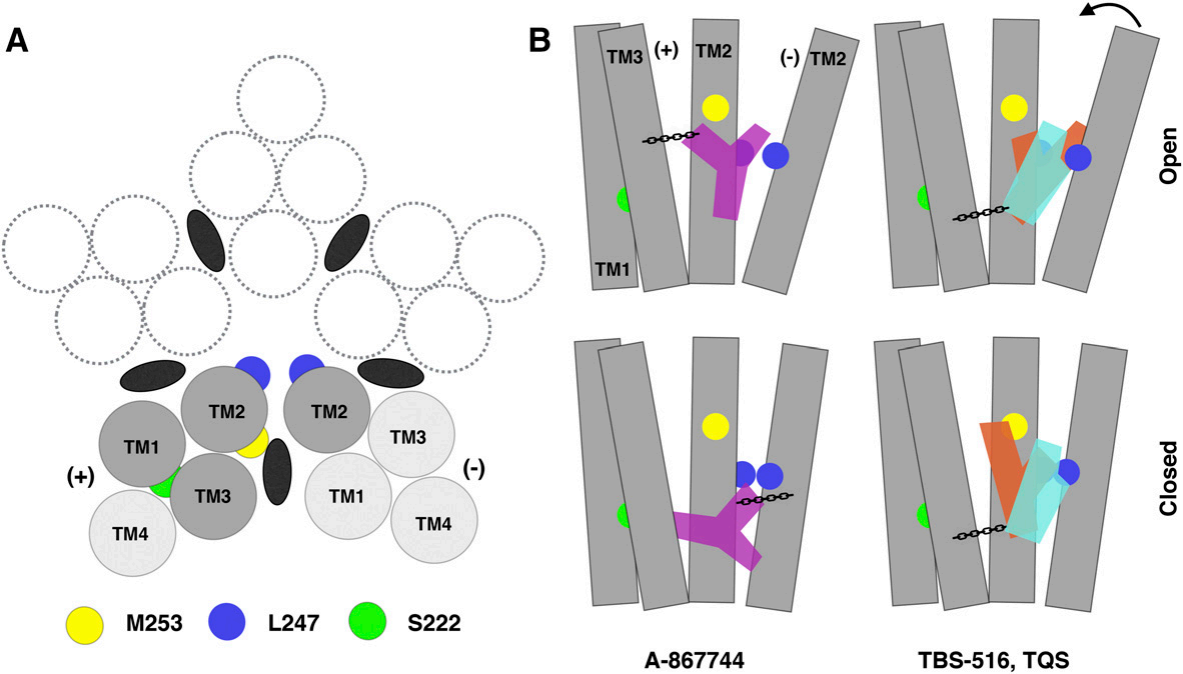
Intersubunit transmembrane binding site for PAMs on the *α*7 nAChR. (A) Representation of the transmembrane domain viewed from above, looking down the axis of the channel pore. Black ellipses indicate the location of the intersubunit allosteric binding site identified in this study. (B) Predicted binding modes in the closed and open receptor models of A-867744 (purple), TBS-516 (orange), and TQS (cyan), shown in relation to transmembrane helices (gray rods) from the principal (+) and the complementary (−) subunit interface. The locations of predicted hydrogen bonds are shown with chain links to denote anchoring of the ligands within the binding site. The locations of amino acids M253, L247, and S222 are shown as yellow, blue, and green circles, respectively. An arrow at the top of TM2 from the complementary subunit denotes the motion required for the change in conformation from the open to the closed channel.

It is notable that some of the mutations examined (S222M, L247T, and M253L) convert A-867744 into an antagonist but do not have this effect with either TBS-516 or TQS (Table 1). In the closed conformation, A-867744 is predicted to bind with a moiety protruding between the TM2 and TM3 helices of the principal subunit, while forming a hydrogen bond with the complimentary subunit (Figs. 9 and 10; Supplemental Fig. 7). This may allow A-867744 to act as a physical barrier to the normal movement of transmembrane helices. It is possible that some transmembrane mutations may increase the energy barrier for transition between closed and open states when A-867744 is bound, causing the observed inhibition of agonist-evoked responses. Alternatively, A-867744 may be acting as an open-channel blocker on receptors containing some transmembrane mutations. However, evidence that other PAMs can block the inhibitory effect of A-867744 (Fig. 5C) provides support for the conclusion that positive and negative modulatory effects can occur through a broadly similar mutually exclusive binding site.

In summary, evidence has been obtained from *α*7 nAChRs containing five different point mutations indicating that A-867744 exerts its allosteric effects via a mechanism that is distinct from other type II PAMs. Docking simulations in the transmembrane regions of new structural models of the human *α*7 nAChRs suggest how PAMs such as A-867744, TBS-516, and TQS interact with an intersubunit transmembrane site and provide an explanation of the pharmacological diversity among this group of chemically similar allosteric modulators.

## Abbreviations

*A-585539*: *(1*S*,4*S*)-2,2-dimethyl-5-(6-phenylpyridazin-3-yl)-5-aza-2-azoniabicyclo[2.2.1]heptane*
*A-867744*: *4-(5-(4-chlorophenyl)-2-methyl-3-propionyl-1*H*-pyrrol-1-yl)benzenesulfonamide*
*α*-BTX: *α*-bungarotoxin
cryo-EM: electron cryo-microscopy
MLA: methyllycaconitine
nAChR: nicotinic acetylcholine receptor
PAM: positive allosteric modulator
PDB ID: protein data bank identifier
pLGIC: pentameric ligand-gated ion channel
RMSD: root mean square deviation
TBS-516: 4-(5-benzyl-3-(4-bromophenyl)-1*H*-1,2,4-triazol-1-yl)benzenesulfonamide
TM: transmembrane
TQS: *cis*-*cis*-4-(napthalen-1-yl)-3*a*,4,5,9*b*-tetrahydro-3*H*-cyclopenta[*c*]quinoline-8-sulfonamide
